# Structural Stability of Silicone-Based Elastodontic Appliances After Clinical Use: Insights from FTIR Spectroscopy

**DOI:** 10.3390/ma19010013

**Published:** 2025-12-19

**Authors:** Emilia-Brindusa Brăilă, Vlad Tiberiu Alexa, Stefania Dinu, Vanessa Bolchis, Vlase Titus, Vlase Gabriela, Atena Galuscan, Daniela Jumanca

**Affiliations:** 1 Doctoral School, ‘Victor Babes’ University of Medicine and Pharmacy Timisoara, 300041 Timisoara, Romania; braila.emilia@umft.ro; 2 Translational and Experimental Clinical Research Centre in Oral Health, Faculty of Dental Medicine, “Victor Babes” University of Medicine and Pharmacy Timisoara, 300041 Timisoara, Romania; vlad.alexa@umft.ro (V.T.A.); vanessa.bolchis@umft.ro (V.B.); galuscan.atena@umft.ro (A.G.); jumanca.daniela@umft.ro (D.J.); 3 Clinic of Preventive, Community Dentistry and Oral Health, Faculty of Dental Medicine, “Victor Babes” University of Medicine and Pharmacy Timisoara, 300041 Timisoara, Romania; 4 Department of Pediatric Dentistry, Faculty of Dental Medicine, “Victor Babes” University of Medicine and Pharmacy Timisoara, 300041 Timisoara, Romania; 5 Pediatric Dentistry Research Center, Faculty of Dental Medicine, Victor Babes University of Medicine and Pharmacy, 300041 Timisoara, Romania; 6 Research Centre for Thermal Analysis in Environmental Problems (ICAM), West University of Timisoara, 300115 Timisoara, Romania; titus.vlase@e-uvt.ro (V.T.); gabriela.vlase@e-uvt.ro (V.G.)

**Keywords:** elastodontic appliances, FTIR spectroscopy, surface modification, structural stability, clinical performance, orthodontics, dental materials

## Abstract

*Background and Objectives:* Elastodontic appliances made of medical-grade silicone are increasingly used in interceptive orthodontics, but prolonged intraoral exposure may affect their stability. This study evaluated structural changes in LM-Activator^TM^ 2 appliances after clinical use, using Fourier-transform infrared (FTIR) spectroscopy. *Materials and Methods:* Eight appliances (one unused control and seven worn for 3–24 months) were analyzed by FTIR-ATR in the 4000–650 cm^−1^ range. Absorption bands characteristic of polydimethylsiloxane (PDMS) were quantified, and indices reflecting backbone crosslinking, side-group retention, hydrophilicity, and relative reduction in methyl-related spectral contributions were calculated. *Results:* The PDMS backbone remained chemically intact across all samples. However, progressive molecular reorganization was detected with wear duration. The Backbone Dominance Index increased significantly from control to 24 months, while side-group indices decreased, confirming apparent depletion of methyl-related FTIR bands. Hydrophilicity and crosslinking indices rose over time, particularly after 12 months, indicating increased surface polarity and network densification. *Conclusions:* LM-Activator^TM^ 2 appliances undergo gradual intraoral aging, marked by backbone crosslinking and apparent reduction in methyl-associated vibrational contributions inferred from FTIR ratio side-groups. These changes, while not compromising the polymer identity, may influence surface properties, biofilm retention, and long-term mechanical behavior. Periodic replacement is recommended to ensure optimal clinical performance.

## 1. Introduction

Oral myofunctional therapy is designed to enhance perioral muscle function and coordination, thereby supporting orthodontic treatment outcomes. By retraining muscle activity, oral myofunctional therapy contributes to the restoration of neuromuscular balance, guides dentoalveolar development, and can influence facial growth patterns [1,2,3,4,5].

One of the commonly used devices in oral myofunctional therapy is the elastodontic appliance (EA), a prefabricated and flexible device manufactured from medical-grade silicone [6]. These appliances are specifically designed to combine functional rehabilitation with orthodontic effects [7]. The LM-Activator^TM^ 2 (LM-Dental, Parainen, Finland) represents a widely used EA that facilitates tooth eruption, improves occlusal relationships, and supports the correction of oral muscle function [8,9].

Patients are typically instructed to wear the LM-Activator^TM^ 2 during the night in addition to several hours during the day, with an average daily use of approximately 12 h [10]. This prolonged intraoral exposure subjects the device to multiple challenges, including mechanical loading, temperature fluctuations, variations in salivary pH, and microbial colonization. Over time, these conditions may lead to structural and surface modifications of the appliance, potentially compromising its mechanical properties and functional efficiency [11,12,13,14].

Previous studies show that intraoral devices made from silicone and related polymers experience aging processes marked by increased surface roughness, water sorption, and chemical and mechanical degradation. For example, Invisalign and 3D-printed aligners exhibit significantly rougher surfaces after intraoral exposure [15]; elastodontic appliances like the LM-Activator^TM^ 2 display filler-particle delamination and reduced viscoelasticity following intraoral and artificial aging [14]. These changes can foster microbial adhesion, reduce mechanical stability, and impair therapeutic efficacy. Increased biofilm formation and microbial colonization have been demonstrated on different clear aligner systems after intraoral use [16]. Additionally, 3D-printed and thermoformed aligners show compromised chemical and color stability under aging conditions [17] while in-house 3D-printed aligners lose mechanical strength over time in vivo [18]. Systematic reviews further confirm consistent evidence of structural and functional degradation across appliance types [19]. While such degradative patterns are well-established in aligners and removable orthodontic appliances, evidence specific to elastodontic appliances remains scarce, underscoring the importance of evaluating long-term structural stability of devices like the LM-Activator™ 2 (LM-Dental AB, Helsingborg, Sweden) for reliable clinical practice.

The aim of this study was to investigate relative intraoral aging trends and molecular level spectral changes in silicone-based elastodontic appliances (LM-Activator^TM^ 2) after different periods of intraoral use (3–24 months), using FTIR-ATR spectroscopy and derived indices reflecting PDMS backbone dominance, side-group retention, and network reorganization. The null hypothesis was that intraoral service duration does not induce significant differences in PDMS-related FTIR spectral features or FTIR-derived indices when compared with the unused control specimen.

## 2. Materials and Methods

### 2.1. Sample Selection and Preparation

**Figure 1:**
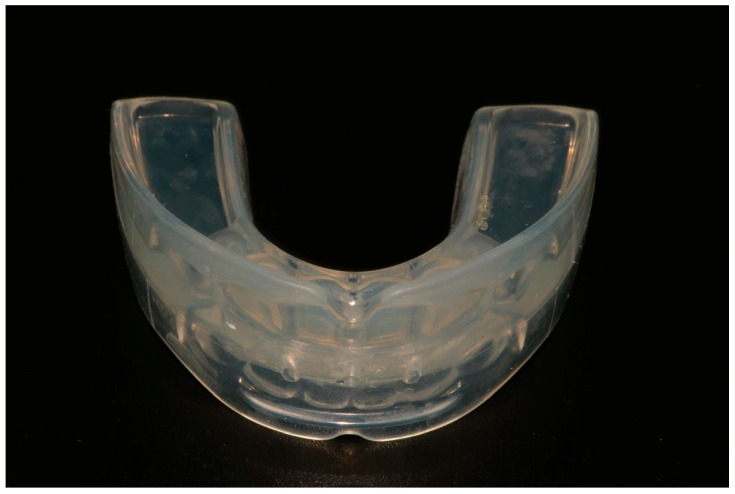
LM-Activator^TM^ 2, LM-Dental, Parainen, Finland.

This study evaluated structural modifications in elastodontic appliances made of medical-grade silicone, specifically polydimethylsiloxane (PDMS) LM-Activator™ 2 (LM-Dental AB, Helsingborg, Sweden) after different durations of intraoral use (Figure 1).

A total of eight appliances were included: one control specimen (not exposed to intraoral conditions) and seven appliances worn by patients. The unequal number of appliances available for the different wear-duration groups reflects routine clinical practice and patient-specific treatment timelines, as appliances were collected retrospectively after completion of their prescribed intraoral use. All specimens were analyzed under identical experimental conditions, and comparisons focused on consistent intra-group trends rather than on balanced group sizes. All patients were instructed to wear the appliance overnight and for additional daytime hours, totaling approximately 12 h per day. After the designated treatment period ranging from 3 to 24 months of intraoral use, the appliances were collected from patients during routine orthodontic visits. Each appliance was immediately rinsed with distilled water to remove saliva residues, air-dried at room temperature, and stored individually in sterile, airtight containers. For analytical purposes, each appliance was sectioned into fragments of approximately 10 × 10 mm. This procedure yielded a total of 71 individual specimens, which were used for subsequent FTIR measurements as presented in Table 1. Samples were labeled according to the duration of wear and subsequently transported under dry conditions to the Advanced Environmental Research Institute (ICAM, West University of Timișoara, Romania) for laboratory analyses.

### 2.2. Fourier-Transform Infrared Spectroscopy (FTIR)

Attenuated Total Reflectance Fourier-Transform Infrared (FTIR-ATR) spectroscopy was performed at room temperature using a PerkinElmer Spectrum 100 spectrometer (PerkinElmer, Waltham, MA, USA). Spectra were recorded in the 4000–650 cm^−1^ range, with a resolution of 1 cm^−1^ and 16 co-added scans per measurement. The resulting spectra were analyzed for characteristic absorption bands to detect potential chemical modifications and molecular structural changes induced by intraoral aging [20].

### 2.3. FTIR-Derived Parameters

To further interpret the FTIR spectra, corrected absorbance areas of principal PDMS bands were extracted for each specimen:

– Si–O–Si stretching (~1006–1008 cm^−1^);
– Si–CH_3_ deformation (~1257 cm^−1^);
– CH_3_ stretching (~2962 cm^−1^);
– Si–C/CH_3_ rocking (~790–800 cm^−1^).

From these values, the following additional indices were calculated:

Backbone Dominance Index (BDI) indicates crosslinking and network densification in the polymer backbone, often occurring with aging and repeated mechanical/thermal stress. A higher value means the PDMS siloxane backbone (Si–O–Si) is more prominent relative to the methyl side-groups (Si–CH_3_). BDI is calculated with formula:

(1) $$BDI=\;\frac{Si–O–Si\;band\;area}{Si–{CH}_{3}\;band\;area}$$

Side-Group Retention Index (SGRI). A lower value means there are fewer methyl side-groups relative to the backbone. A decrease suggests side-chain scission or rearrangement, potentially leading to a more hydrophilic surface that could favor plaque and biofilm accumulation. It is calculated with the formula:

(2) $$SGRI=\;\frac{{CH}_{3}\;stretch\;band\;area\;}{Si–O–Si\;band\;area}$$

Rocking/Backbone Ratio (RBR) measures the relative stability of the rocking mode compared to the backbone. It is less sensitive to degradation and is therefore useful as a robust reference against variability in ATR contact or sample thickness.

(3) $$RBR=\frac{Si–C/{CH}_{3}\;rocking\;band\;area\;(\sim 790-800\;{cm}^{-1})\;}{Si–O–Si\;band\;area}$$

Hydrophilicity Index (HPI) calculated with the formula:

(4) $$HPI=\;\frac{Area(Si–O–Si)}{Area({CH}_{3}\;stretch)}$$

A higher HPI indicates fewer hydrophobic methyl groups per siloxane backbone unit, implying increased surface polarity.

Crosslinking Index (CLI). An increased CLI suggests densification and crosslinking of the silicone network.

(5) $$CLI=\frac{Area(Si–O–Si)\;[+Area(Rocking)]}{Area(Si–{CH}_{3})}$$

Methyl Loss Index (MLI) is calculated with the formula:

(6) $$MLI=\;\frac{1}{HPI}$$

A decreasing MLI reflects progressive methyl group depletion over time.

It should be emphasized that the FTIR-derived indices introduced above are empirical, semi-quantitative descriptors derived from ratios of integrated absorbance bands within the same spectrum, rather than absolute measurements of bond concentration or elemental composition. Because FTIR band intensity depends on both the number of oscillators and the magnitude of the dipole-moment change during vibration, these metrics are interpreted as indicators of relative spectral reorganization associated with wear duration, measured under identical ATR-FTIR conditions. Their purpose is to highlight systematic intra-group trends rather than provide quantitative bond or elemental ratios.

The selection of the specific FTIR bands used for index construction was based on band specificity, spectral stability, and reproducibility in PDMS-based materials. The narrow range around 1006–1008 cm^−1^ was chosen to represent the Si–O–Si asymmetric stretching vibration because it corresponds to the most intense and well-defined backbone-related absorption maximum in PDMS, while minimizing overlap with adjacent siloxane vibrational modes present in the broader 850–1100 cm^−1^ region. The wider region contains multiple overlapping Si–O–Si stretching and bending contributions and is more sensitive to baseline variations, making it less suitable for consistent quantitative comparison across samples. Conversely, although the ~1400 cm^−1^ band is associated with CH_3_ deformation modes, this region is particularly susceptible to spectral overlap with adsorbed organic residues and environmental contributions in intraorally exposed specimens. For this reason, it was considered suitable for qualitative observation but was excluded from quantitative index construction to improve robustness and specificity of the derived metrics.

These parameters were calculated for all sections corresponding to the specimens listed in Table 1 and are presented as mean values ± standard deviation.

### 2.4. Statistical Analysis

The results are presented as mean values ± standard deviation (SD). The number of replicates for each sample was 10 for specimens P1–P7 and 2 for the control specimen P0. Differences between means were evaluated using one-way ANOVA, followed by multiple comparison analysis performed with a *t*-test (two samples, assuming equal variances) in Microsoft Excel 365. Statistical significance was set at *p* < 0.05.

### 2.5. Ethical Approval

The study protocol was approved by the Ethics Committee of Scientific Research of the “Victor Babeș” University of Medicine and Pharmacy Timișoara, Romania (approval code: Aviz CECS al UMFTVB Nr. 85/01.11.2021). The elastodontic appliances analyzed in this study were obtained after clinical use in pediatric patients aged 6–12 years. Written informed consent was obtained from the patients’ legal guardians prior to appliance collection, and all samples were anonymized before analysis. The study was conducted in accordance with the principles of the Declaration of Helsinki.

## 3. Results

### 3.1. FTIR-Derived Structural Changes After Intraoral Aging

**Figure 2:**
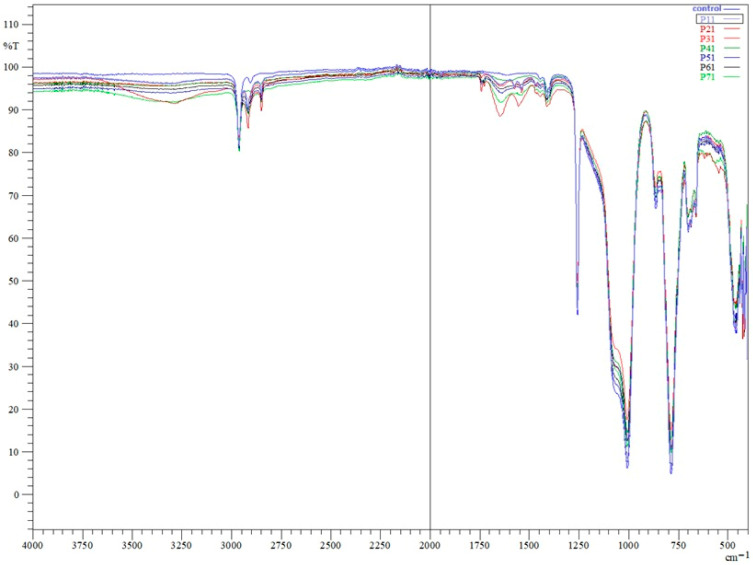
The FTIR spectra are presented for one representative fragment corresponding to each analyzed specimen, as listed in Table 1.

Fourier-transform infrared (FTIR) analysis of the control appliance (P0) and appliances worn for 3 months (P6), 6 months (P3, P4, P5), 12 months (P1, P2), and 24 months (P7) revealed the characteristic absorption profile of polydimethylsiloxane (PDMS). Across all spectra, the main PDMS bands were present: Si–O–Si stretching in the ~1006–1008 cm^−1^ region, Si–CH_3_ deformation near 1257 cm^−1^, CH_3_ stretching around 2962 cm^−1^, and Si–C/CH_3_ rocking at ~790–800 cm^−1^. The consistent presence of these bands in all samples confirms that the fundamental PDMS backbone remained chemically intact over the appliance’s service life, regardless of wear duration. No new peaks indicative of oxidative degradation, such as carbonyl groups in the ~1700–1800 cm^−1^ region, were detected in the worn samples (Figure 2). However, additional absorption features were observed in worn appliances, including a broad band around ~3400 cm^−1^, changes in the 2750–2900 cm^−1^ region, and bands near ~1600 cm^−1^ and ~1400 cm^−1^, which became more evident with increasing wear duration.

The broad feature observed around ~3400 cm^−1^ is consistent with O–H stretching vibrations, while the feature near ~1600 cm^−1^ is non-specific and may reflect overlapping contributions from absorbed water and/or adsorbed organic species; therefore, it is interpreted qualitatively rather than used for quantitative inference [21].

The 2750–2900 cm^−1^ region comprises symmetric and asymmetric C–H stretching vibrations of methyl groups characteristic of PDMS, and the ~1400 cm^−1^ region is associated with CH_3_ deformation modes. These features were absent or markedly less pronounced in the unused control specimen and became progressively more evident in samples subjected to longer intraoral exposure.

#### 3.1.1. Quantitative Spectral Changes with Wear Duration

Analysis of corrected peak areas revealed a progressive increase in the Si–O–Si band area with longer wear durations, particularly in the 12-month and 24-month samples, consistent with backbone crosslinking and densification of the silicone network. In parallel, a reduction in the relative intensity of Si–CH_3_ and CH_3_ stretching bands was observed over time, most pronounced in P7, suggesting side-chain scission and a loss of hydrophobic methyl groups. The ~790–800 cm^−1^ rocking region showed only slight decreases in relative area, indicating lower sensitivity to wear-related chemical changes.

#### 3.1.2. Group-Specific Differences

For the 6-month group (P3, P4, P5), the calculated mean ± standard deviation values were BDI: 25.13 ± 0.19, SGRI: 0.03 ± 0.00, and RBR: 0.36 ± 0.00, representing intermediate values between the control and longer wear durations, consistent with early but measurable structural reorganization. For the 12-month group (P1, P2), the mean ± standard deviation values were BDI: 26.81 ± 0.27, SGRI: 0.03 ± 0.00, and RBR: 0.35 ± 0.00, indicating more pronounced backbone/methyl shifts.

#### 3.1.3. Spectral Indices and Correlations

In Table 2 the value of indices of FTIR derived parameters (BDI, SGRI and RBR) are presented. It can be observed that BDI increased and SGRI decreased significantly with wear duration (*p* < 0.05), while RBR exhibited only a mild negative trend.

BDI increased steadily from 23.05 (P0, control) to 28.95 (P7, 24 months). This confirms a progressive dominance of the siloxane backbone with wear time, consistent with intraoral-induced crosslinking of the PDMS network. The largest jumps occur after 12 months, showing that long-term use accelerates these structural changes. SGRI remained low (~0.03) but showed a slight decline by 24 months, supporting the gradual apparent reduction in methyl-associated vibrational contributions inferred from FTIR ratio side-groups over time, which might subtly increase surface polarity and the potential for microbial adhesion. RBR decreased modestly from 0.37 (P0) to 0.34 (P7), indicating that this vibrational mode is relatively unaffected by the degradation processes, confirming its suitability as a stable internal reference when normalizing ATR-FTIR data.

Table 3 presents the correlation matrix between wear duration and FTIR-derived indices (BDI, SGRI and RBR).

The correlation matrix reveals very strong positive correlations between wear duration and BDI (r = 0.98), and strong negative correlations between wear duration and SGRI (r = –0.97). This confirms that as intraoral service time increases, the siloxane backbone becomes more dominant while methyl side-groups diminish. BDI and SGRI are almost perfectly inversely related (r = –0.99), reinforcing that both parameters reflect the same underlying structural changes in the PDMS network. RBR shows only a moderate negative correlation with wear duration (r = –0.54), indicating relative stability and supporting its use as a robust ATR normalization reference. The moderate correlation between SGRI and RBR (r = 0.55) suggests minor concurrent changes, though not as pronounced as those seen in backbone or methyl indices.

**Figure 3:**
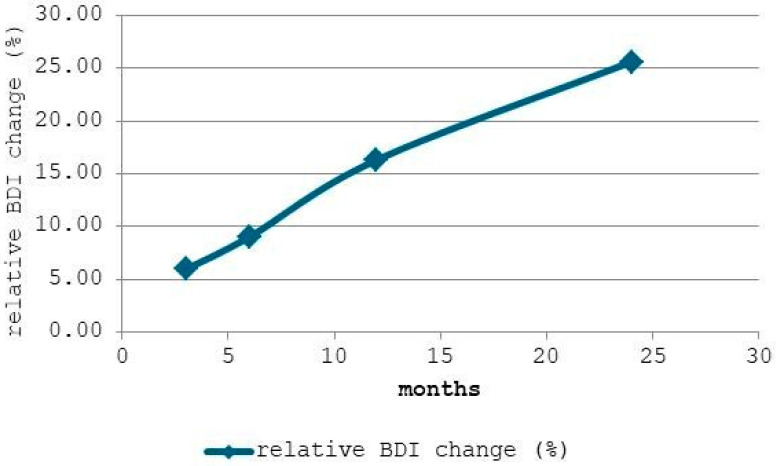
Relative Increase in BDI with Wear Duration.

Figure 3 displays the relative change in the Backbone Dominance Index (BDI) compared to the baseline (0 months). It can be observed that after 3 months, BDI increased by approximately 5.95%, indicating that crosslinking processes and structural rearrangements in the PDMS network begin relatively early in intraoral service. At 6 months, the relative change reached 9.02%, showing a sustained but moderate progression, while after 12 months, the BDI had increased by 16.38%, suggesting a more pronounced alteration of the siloxane backbone relative to methyl side-groups.

The steady increase, particularly after 12 months, confirms that intraoral aging promotes progressive dominance of the siloxane backbone, likely due to crosslinking and reduced chain mobility (i.e., reduced rotational and translational freedom of PDMS polymer segments between crosslinks). This structural evolution may translate into increased rigidity, reduced elasticity, and potential changes in clinical performance. At 24 months, the change peaked at 25.53%, reflecting a substantial reorganization of the polymer network and confirming the accelerated backbone densification phase in prolonged wear.

Overall conclusion: The FTIR indices clearly indicate that LM-Activator^TM^ 2 appliances undergo measurable molecular reorganization during intraoral service. The main process appears to be crosslinking of the PDMS backbone, accompanied by a gradual reduction in methyl side-groups. While these changes do not alter the fundamental polymer identity, they likely affect mechanical properties (e.g., stiffness, viscoelasticity) and surface interactions (e.g., plaque retention potential) after prolonged wear.

### 3.2. Influence of Saliva and Oral Microbiota on FTIR-Derived Parameters

In addition to the previously reported BDI, SGRI, and RBR, three supplementary indices were calculated to better capture the molecular-level changes driven by the oral environment: HPI, CLI and MLI (Table 4).

The FTIR spectral data demonstrated that the LM-Activator^TM^ 2 appliances maintained their characteristic PDMS backbone signals (Si–O–Si, Si–CH_3_, CH_3_ stretch, and rocking modes) throughout all wear durations, indicating that the core silicone network remained chemically intact. However, quantitative analysis of absorbance ratios revealed progressive changes in key indices that are strongly associated with intraoral environmental effects, including prolonged exposure to saliva and biofilm activity.

Hydrophilicity Index (HPI) increased progressively with wear duration, reflecting a gradual loss of hydrophobic methyl groups and a shift toward higher surface polarity. This is consistent with side-chain scission induced by enzymatic activity and acidic/oxidative byproducts of oral microbiota.

Crosslinking Index (CLI) showed a marked increase over time, indicating densification of the silicone network, which may result from long-term chemical stress in the oral cavity (pH fluctuations, ionic exposure, oxidative metabolites).

Methyl Loss Index (MLI) decreased steadily with wear time, further confirming the progressive depletion of methyl side-groups.

Quantitatively, HPI values increased from 31.25 (P0) to 45.20 (P7), while CLI rose from 30.73 (P0) to 59.38 (P7). These trends were most pronounced after 12 and 24 months, aligning with the period when biofilm maturation and sustained biochemical activity are expected to exert greater cumulative effects. The stability of RBR values across all durations indicates that these changes are primarily chemical rather than artifacts of ATR-FTIR measurement conditions.

Taken together, these findings provide strong evidence that saliva and oral microbiota contribute significantly to the molecular reorganization of LM-Activator^TM^ 2 materials during clinical use. The combined increase in backbone dominance, hydrophilicity, and crosslinking over time supports the hypothesis that intraoral aging is driven by synergistic mechanical, chemical, and microbiological factors.

The correlation analysis of the additional FTIR-derived parameters revealed several strong and biologically relevant relationships (Table 5). Strong positive correlation between HPI and CLI (r > 0.95) which indicates that increased hydrophilicity, driven by the apparent reduction in methyl-associated vibrational contributions inferred from FTIR ratios, is closely associated with increased network crosslinking. Both effects are consistent with long-term chemical modification of the PDMS matrix under intraoral exposure to saliva and microbial metabolites.

Strong negative correlation was observed between HPI and MLI (r ≈ –1.00). Since MLI is the inverse of HPI, this result confirms their mathematical relationship and validates the internal consistency of the dataset. The strong inverse link also reinforces that apparent depletion of methyl-related FTIR bands is directly tied to the observed hydrophilicity increase.

Moderate positive correlation between CLI and RBR suggests that while the rocking mode is relatively stable, small concurrent changes occur alongside network densification, potentially due to alterations in local bond dynamics within the silicone elastomer.

Duration-related trends—Both HPI and CLI exhibit strong positive correlations with wear duration (r > 0.9), supporting that hydrophilicity increase and crosslinking intensification are cumulative processes over time. In contrast, MLI correlates strongly and negatively with wear duration, confirming progressive relative reduction in methyl-associated FTIR contributions during intraoral service.

Overall, the correlation structure supports the interpretation that oral biofilm activity and saliva chemistry act synergistically to enhance backbone crosslinking and surface polarity, while depleting hydrophobic methyl groups. These changes may have functional implications for the appliance’s long-term mechanical resilience and surface biofilm interactions.

## 4. Discussion

The progressive spectral changes observed in LM-Activator^TM^ 2 appliances suggest a direct influence of the oral environment—particularly saliva and resident microbiota—on the material’s molecular structure during intraoral service. Across all wear durations, the characteristic PDMS bands (Si–O–Si stretching, Si–CH_3_ deformation, CH_3_ stretching, and Si–C/CH_3_ rocking) were preserved, indicating that the polymer backbone remained chemically intact. However, the consistent increase in Si–O–Si band area and reduction in methyl-related intensities (Si–CH_3_ and CH_3_ stretch) were most pronounced after 12–24 months, supporting a mechanism of side-chain scission coupled with backbone densification/crosslinking—aging pathways commonly captured by FTIR in silicone systems [22,23]. These trends align with prior FTIR-based analyses showing crosslinking/oxidation signatures and reduced side-group content after aging in silicone elastomers and aligner polymers [14,24,25]. These interpretations rely on consistent intra-group trends and strong correlations across all specimens rather than on absolute intensity values, thereby reducing the influence of random conformational variability on the observed spectral changes.

Functionally, these chemical changes can shift the viscoelastic behavior of the material toward greater stiffness and altered stress-relaxation patterns. Comparable effects have been reported in both in vitro and in vivo studies on aligner and retainer polymers, where water sorption and thermocycling reduce mechanical properties, thermoforming alters microstructure and thickness, and aging leads to tensile strength deterioration [26,27,28,29,30]. Collectively, these findings support the clinical observation that prolonged appliance service compromises force delivery and surface integrity.

Salivary conditions further modulate degradation. Water ingress (plasticization) and pH excursions can accelerate network rearrangement and side-group depletion, increasing surface polarity—an effect mirrored by our higher backbone-weighted indices (e.g., BDI/CLI) and lower side-group metrics (e.g., SGRI/MLI). Experimental work links water sorption to topographical and optical changes in PETG appliances, while pH-dependent aging of elastodontic silicone shows greater side-chain scission in alkaline media—mechanistically congruent with our FTIR-derived shifts.

From a conceptual perspective, the FTIR-derived changes observed in this study can be visualized as a progressive transition from a flexible PDMS network with mobile siloxane chains and methyl-rich side groups toward a denser network characterized by increased backbone dominance, reduced segmental mobility, and enhanced surface-level interactions with water and salivary components. In unused specimens, PDMS chains exhibit greater rotational and translational freedom, whereas prolonged intraoral exposure is associated with network densification and surface hydration or adsorption phenomena. This description is intended as a conceptual visualization of trends inferred from FTIR analysis rather than a representation of specific chemical reactions.

The additional absorption features identified in worn appliances further support the influence of the oral environment on the silicone material. The broad band around ~3400 cm^−1^ and the feature near ~1600 cm^−1^ are consistent with O–H stretching and H–O–H bending vibrations, respectively, and are commonly associated with absorbed water and hydrogen-bonded species in PDMS-based materials exposed to humid or aqueous environments [14,24]. Similar FTIR signatures have been reported for silicone elastomers and orthodontic polymers following water immersion or intraoral aging, reflecting surface hydration and water uptake rather than oxidative chemical degradation [24,25]. The 2750–2900 cm^−1^ region, corresponding to C–H stretching vibrations of methyl groups, showed subtle variations in band shape and relative intensity that are consistent with changes in local molecular environment and conformational rearrangements induced by long-term exposure to saliva and mechanical stress [14,25]. The ~1400 cm^−1^ band, associated with CH_3_ deformation modes and potential overlap with adsorbed organic residues, was not used for quantitative index construction due to its susceptibility to environmental contributions and spectral overlap. Overall, these features are interpreted as indicators of surface-level hydration and adsorption phenomena accompanying intraoral service, rather than the formation of new chemical species.

Microbiological implications are notable: rougher, more polar surfaces favor early colonizers and biofilm maturation. In line with this, recent in vitro and in vivo studies demonstrate time-dependent biofilm accumulation across aligner materials [31], supporting a practical link between chemical/surface aging and hygiene risk during extended wear [16,32]. From a clinical perspective, increased surface polarity and reduced methyl group content may favor biofilm accumulation, staining, and alterations in appliance flexibility, potentially compromising long-term performance and patient compliance. The findings support the need for regular monitoring and timely replacement of elastodontic appliances to ensure consistent therapeutic efficacy.

Overall, the present findings suggest that multiple intraoral factors contribute cumulatively to the molecular aging of PDMS-based elastodontic appliances. Thermal cycling and repeated mechanical loading may promote network rearrangement and gradual densification, as reflected by the increasing backbone-dominated FTIR indices observed with prolonged use. Continuous exposure to moisture and saliva can further facilitate network reorganization through water ingress and plasticization, while pH fluctuations and biofilm-derived metabolites may accelerate side-group alterations at the material surface. These combined effects become particularly evident beyond 12 months of intraoral service, indicating a time-dependent progression of molecular changes. In the context of the existing literature, these observations support the clinical need for regular monitoring of elastodontic appliances and consideration of timely replacement, as well as the implementation of appropriate cleaning protocols and wear-time guidance to mitigate biofilm-mediated and hydrolytic aging. Future investigations should integrate longitudinal FTIR analysis with complementary mechanical and microbiological assessments on larger patient cohorts to better relate molecular-level changes to long-term clinical performance.

Accordingly, the present study was designed as an FTIR-based spectroscopic investigation aimed at identifying relative intraoral aging trends in clinically retrieved elastodontic appliances. From a methodological perspective, ATR-FTIR cannot fully separate the respective contributions of conformational variability, changes in dipole-moment derivatives, and true chemical or network rearrangements occurring during intraoral aging. Consequently, the FTIR-derived indices used in this study should be regarded as semi-quantitative markers of relative spectral reorganization, rather than definitive quantitative measurements of methyl-group loss, bond concentration, or elemental ratios. Additional surface-sensitive and compositional techniques would be required to definitively confirm these structural interpretations with higher chemical specificity.

It should also be emphasized that the observed variations in methyl-related FTIR indices reflect relative changes in vibrational contributions and local molecular environments, rather than direct quantitative measurements of methyl group concentration or Si/C elemental ratios. Absolute compositional assessment would require complementary surface-sensitive or elemental analysis techniques, such as X-ray photoelectron spectroscopy or related methods. In addition, a comprehensive assessment of structure–property relationships during intraoral aging would require complementary thermal, mechanical, and surface analyses, including differential scanning calorimetry, thermogravimetric analysis, wettability measurements, and mechanical testing (e.g., tensile strength and elongation at break), as reported in recent aging studies of polymeric materials. These analyses were beyond the scope of the present FTIR-focused investigation and are planned for future studies on larger sample cohorts.

## 5. Conclusions

This study demonstrated that LM-Activator^TM^ 2 elastodontic appliances undergo measurable structural changes during intraoral service, as evidenced by FTIR-derived parameters. While the fundamental PDMS backbone remained chemically intact across all wear durations, progressive increases in backbone-dominated indices and crosslinking-related spectral features, together with relative reductions in methyl-associated vibrational contributions, indicate ongoing network reorganization during clinical use. These changes became particularly pronounced after 12 and 24 months, underscoring the cumulative influence of salivary exposure, microbial activity, thermal fluctuations, and mechanical stress inherent to the oral environment.

Although the present investigation was limited by the number of analyzed appliances and relied exclusively on FTIR-based characterization, it provides novel molecular-level insight into the intraoral aging behavior of elastodontic devices. The findings support the need for continued research integrating complementary surface, mechanical, and microbiological analyses on larger cohorts to better elucidate the relationship between molecular aging processes and long-term clinical performance.

## Figures and Tables

**Table 1:** Sample presentation. For clarity, samples are presented according to their original collection codes; the corresponding intraoral wear durations are explicitly indicated in the table.

Sample	Number of Sections	Worn Duration
Control	P0–P1	0
P1	P2–P11	12 months
P2	P12–P21	12 months
P3	P22–P31	6 months
P4	P32–P41	6 months
P5	P42–P51	6 months
P6	P52–P61	3 months
P7	P62–P71	24 months

**Table 2:** FTIR-Derived Indices (Mean ± SD) by Wear Duration. Different superscript letters ^(a–e)^ indicate statistically significant differences between wear-duration groups (*p* < 0.05), as determined by one-way ANOVA followed by pairwise t-tests assuming equal variances.

Wear Duration (Months)	BDI (Mean ± SD)	SGRI (Mean ± SD)	RBR (Mean ± SD)
0 (Control)	23.05 ± 0.00 ^a^	0.03 ± 0.00 ^a^	0.37 ± 0.00 ^a^
3	24.42 ± 0.00 ^b^	0.03 ± 0.00 ^a^	0.36 ± 0.00 ^b^
6	25.13 ± 0.19 ^c^	0.03 ± 0.00 ^a^	0.36 ± 0.00 ^bc^
12	26.81 ± 0.27 ^d^	0.03 ± 0.00 ^a^	0.35 ± 0.00 ^d^
24	28.95 ± 0.00 ^e^	0.03 ± 0.00 ^a^	0.34 ± 0.00 ^e^

**Table 3:** Correlation matrix between wear duration and FTIR-derived indices; only the upper triangular portion is shown, with duplicated values and diagonal self-correlations omitted for clarity.

	Duration (Months)	BDI	SGRI	RBR
Duration (months)	—	0.98	−0.97	−0.54
BDI	—	—	−0.99	−0.57
SGRI	—	—	—	0.55
RBR	—	—	—	—

**Table 4:** FTIR-derived Parameters Related to Oral Microflora Influence. Different superscript letters ^(a–h)^ indicate statistically significant differences between groups (*p* < 0.05), as determined by one-way ANOVA followed by pairwise t-tests assuming equal variances.

Sample	Si_O_Si	Si_CH_3_	CH_3__Stretch	Rocking	HPI	CLI	Methyl Loss Index
P0	10,332.47 ^a^	448.48 ^a^	330.56 ^a^	3832.45 ^a^	31.26 ^a^	31.58 ^a^	0.03 ^a^
P1	1751.01 ^b^	484.98 ^b^	235.22 ^b^	3770.54 ^b^	7.44 ^b^	11.39 ^b^	0.13 ^b^
P2	1688.78 ^c^	436.98 ^c^	176.31 ^c^	3478.23 ^c^	9.58 ^c^	11.82 ^c^	0.1 ^c^
P3	8934.61 ^d^	438.64 ^d^	199.57 ^d^	3410.87 ^d^	44.77 ^d^	28.14 ^d^	0.02 ^d^
P4	1476.78 ^e^	457.18 ^e^	205.35 ^e^	3491.45 ^e^	7.19 ^e^	10.87 ^e^	0.14 ^e^
P5	1663.72 ^f^	457.18 ^ef^	205.35 ^ef^	3533.61 ^f^	8.1 ^f^	11.37 ^bf^	0.12 ^f^
P6	9836.72 ^g^	531.98 ^g^	241.34 ^g^	3689.87 ^g^	40.76 ^g^	25.43 ^g^	0.02 ^dg^
P7	9507.79 ^h^	442.28 ^h^	210.41 ^h^	3608.7 ^h^	45.19 ^h^	29.66 ^h^	0.02 ^dh^

**Table 5:** Correlation matrix of FTIR-derived parameters related to oral microflora influence; only the upper triangular portion is shown, with duplicated values and diagonal self-correlations omitted for clarity.

	Si–O–Si	Si–CH_3_	CH_3_ Stretch	Rocking	HPI	CLI	Methyl Loss Index
Si–O–Si	—	0.13	0.52	0.31	0.95	0.99	−0.97
Si–CH_3_	—	—	0.21	0.45	0.08	−0.03	−0.06
CH_3_ stretch	—	—	—	0.84	0.23	0.52	−0.35
Rocking	—	—	—	—	0.07	0.28	−0.16
HPI	—	—	—	—	—	0.93	−0.97
CLI	—	—	—	—	—	—	−0.96
Methyl Loss Index	—	—	—	—	—	—	—

## Data Availability

The original contributions presented in this study are included in the article. Further inquiries can be directed to the corresponding author.
